# Diagnostic tips for *multi-phase post-mortem computed tomography angiography* interpretation in upper gastro-intestinal bleeding

**DOI:** 10.1007/s00414-025-03593-0

**Published:** 2025-09-08

**Authors:** Kim Wiskott, Virginie Magnin, Coraline Egger, Ruben Soto, Silke Grabherr, Tony Fracasso

**Affiliations:** 1 University Center of Legal Medicine Lausanne-Geneva, University of Geneva, Geneva University Hospitals, Rue Michel-Servet 1, 1211 Geneva 4, Switzerland; 2 University Center of Legal Medicine Lausanne-Geneva, University of Lausanne, Lausanne University Hospital, Chemin de La Vuillette 4, 1000 Lausanne, Switzerland

**Keywords:** Upper gastro-intestinal bleeding, Gastro-intestinal hemorrhages, Post-mortem hemorrhages, Post-mortem angiography, MPMCTA

## Abstract

In the past 10 years, the Multi-phase Post-mortem Computed Tomography Angiography (MPMCTA) has considerably improved the quality and precision of postmortem diagnoses, particularly in cases with vascular implication. MPMCTA is known to have higher sensitivity for detecting the source of a hemorrhage than autopsy. Death by upper gastro-intestinal (GI) bleeding is not so uncommon in forensic practice. MPMCTA, like any other diagnostic test, can produce artifacts that must be recognized. Radiologists at our center have previously encountered images suggestive of upper GI bleeding that were ultimately identified as artifacts during autopsy. This is why we believe it is essential to establish criteria to differentiate true bleeding from artifacts. The aim of our study was to compare the diagnostic value of MPMCTA and autopsy in detecting and localizing sources of upper GI bleeding, and to establish diagnostic criteria to aid in the interpretation of upper GI contrast extravasation on angiography. We conducted a retrospective cross-sectional study, analyzing MPMCTA and autopsy data from 326 human bodies. In the GI tract, contrast extravasation should not be immediately interpreted as a sign of active bleeding. In cases of true GI hemorrhage, MPMCTA reveals specific features suggestive of bleeding, such as hyperdense material within the GI tract on native CT, and a focal contrast leakage during the arterial or venous phase. This article offers tips that may help radiologists and forensic pathologists distinguish true bleeding from artifacts when interpreting MPMCTA findings in the upper GI tract.

## Introduction

Over the past decade, Multi-phase Post-mortem Computed Tomography Angiography (MPMCTA) has significantly enhanced the quality and precision of postmortem diagnoses, especially in cases involving vascular pathology. MPMCTA has been shown to have greater sensitivity than autopsy in identifying the source of hemorrhage [1, 2]. Indications for MPMCTA mainly include vascular lesions, cardiovascular pathologies and sudden cardiac deaths [1–3]. The Lausanne-Geneva University Center of Legal Medicine applies a standardized MPMCTA protocol consisting of one native (without contrast) phase and three angiographic phases using an oily contrast-agent mixture consisting of Paraffin oil and Angiofil® [1, 2]. These four distinct phases provide more accurate radiological documentation and interpretation, reducing the risk of misdiagnosis due to technical artifacts, particularly those caused by residual blood.

The native phase is essential to avoid false-positive findings by identifying preexisting hyperdensities in the gastrointestinal (GI) tract. The subsequent arterial and venous phases, performed with high-density contrast and thin-slice acquisitions, enable the detection of arterial and venous extravasations. Finally, a dynamic phase is conducted: additional contrast agent is injected while scanning is performed simultaneously, allowing for confirmation or refutation of suspected findings from the earlier phases.

The incidence of upper GI bleeding ranges from 40 to 150 episodes per 100,000 persons per year, with a mortality rate of 5% to 14%, increasing up to 40% in high-risk patients [4–10]. Patients typically present with hematemesis and/or melena, and in cases of large-volume bleeding, hematochezia may also occur. Initial evaluation includes assessment of hemodynamic stability and resuscitation if necessary, as well as physical examination and laboratory testing. Early upper endoscopy (within 24 h) remains the clinical gold standard for both the diagnosis and treatment of upper GI bleeding [11, 12]. However, Multi-phase Computed Tomography Angiography has been proposed as an efficient initial diagnostic tool due to its widespread availability and rapid acquisition, which can facilitate prompt diagnosis and management [5, 13–15].

Upper GI bleeding occurred from an injury located proximally to the ligament of Treitz [1]. The causes of upper GI bleeding are typically classified as either variceal—most often in the context of portal hypertension, such as esophageal varices—or non-variceal. Non-variceal causes include peptic ulcers, esophageal lesions (e.g., due to gastroesophageal reflux disease or Mallory-Weiss tears caused by vomiting), external trauma, vascular anomalies (such as angiodysplasia or Dieulafoy lesions), and benign or malignant tumors [5, 16]. In this context, death due to upper GI bleeding is not uncommon in forensic practice, as the circumstances surrounding such bleeding often raise suspicions. Although this may seem high in Western Countries, an Indian study reports that upper GI bleeding accounts 21,3% of all autopsied natural sudden deaths [4].

Postmortem diagnosis of upper GI bleeding remains challenging. The localization of GI bleeding sources may be complicated by artifactual contrast extravasation in the gastric and duodenal mucosa and/or lumen—particularly in cases involving multiorgan failure and/or extended postmortem intervals [17]. Several explanations have been proposed, including postmortem mucosal autolysis of the GI tract, partly due to the proximity of the pancreas [17]. Artifacts appear to affect the upper GI tract more frequently than the lower tract [18].

In recent years, radiologists at our center have encountered numerous cases of GI angiographic extravasation artifacts, which have limited the diagnostic accuracy of MPMCTA in detecting and localizing the true source of GI hemorrhage. In this context, forensic pathologists require clear and reliable guidance for the interpretation of MPMCTA findings prior to autopsy.

The first objective of our study was to compare the diagnostic value of MPMCTA in detecting and localizing sources of upper GI bleeding—with or without fatal outcome—using conventional autopsy as the gold standard. In a second step, radiological images of true and false positives were analyzed to assess whether specific imaging features could help distinguish real bleeding from artifacts. Based on these observations, the second aim of the study was to propose diagnostic criteria to support the interpretation of upper GI contrast extravasation on MPMCTA.

## Materials and methods

A cross-sectional retrospective study was conducted over a two-year period (January 2016 to December 2017) at the Lausanne – Geneva University Center of Legal Medicine (Switzerland). The study was approved by the Geneva Research Ethics Committee (Project ID 2022–02170). During this period, approximately 1,000 autopsies were performed, and MPMCTA was carried out in about one third of the cases (*N* = 332).

All autopsy cases with MPMCTA performed during this time frame were initially included. One case involving an autopsied fetus with angiography (*N* = 1), as well as five cases with technical problems related to angiography (*N* = 5), were excluded.

MPMCTA was performed in the supine position using a LightSpeed VCT 64-slice multidetector computed tomograph (GE Healthcare®, Milwaukee, WI, USA). Following a whole-body native CT scan, MPMCTA was conducted from the vertex to the trochanters according to the standardized protocol [1]. Acquisition parameters were as follows: helical acquisition, 120 kV, 200–400 mA (modulated), 0.8 s rotation time, and slice thickness/interval of 1.25 mm/0.625 mm for the arterial and venous phases, and 2.5 mm/2 mm for the dynamic phase. A lipophilic contrast agent mixture of paraffin oil and Angiofil® was used [1].

Postmortem radiological interpretation was performed jointly by a board-certified radiologist with six years of experience in postmortem imaging and a forensic pathology resident. Cases were categorized radiologically using the four levels of GI contrast extravasation previously described in the literature [17]:

- Grade 0: no extravasation
- Grade 1: mucosal enhancement
- Grade 2: mucosal edema
- Grade 3: edematous mucosal enhancement with intraluminal contrast extravasation

Our analysis focused on grade 3 extravasations in the upper GI tract (esophagus, stomach, duodenum) and the middle GI tract (small intestine). Hemorrhages in these regions are known to be more frequent and potentially fatal. In contrast, lower GI bleeding (colon and rectum) is generally less severe, more chronic, and associated with a lower mortality rate (2–4%); these cases were therefore excluded from the study [19, 20].

There is currently no standardized scientific reference for the radiologic density of gastric contents [21]. In this context, we accepted as suggestive of a hemorrhagic content in a native phase, when the gastric content showed a compact and hyperdense appearance, while recognizing that the mixing of potential hemorrhagic contents with digestive contents requires careful interpretation.

From the autopsy reports, we documented whether a significant quantity (> 20 g) of blood—either fresh or digested—was found in the GI tract, and whether upper GI bleeding was determined to be the cause of death. Additional data collected included demographic and clinical information (sex, age, body mass index [BMI]), delay between death certificate and angiography (then the real post-mortem interval was calculated for the corpses whose time of death could be determined), and the Radiologic Alteration Index (RAI), a measure of postmortem alteration based on gas distribution within the body [22].

Autopsy findings were considered the gold standard. Only grade 3 extravasations were interpreted as true or mimicked GI bleeding.

Statistical analyses were conducted using the Chi-squared test, with a significance threshold set at *p* < 0.05. Analyses were performed using Stata® Intercooled version 16 (StataCorp, College Station, TX, USA).

## Results

### Epidemiologic results

Among the 326 included cases, 71% were male (231/326) and 29% female (95/326). Only four individuals (1.2%) were in the 0–19 years age group. The largest proportion of cases belonged to the 40–59 years group (36.2%, 118/326), followed by the 60–79 years group (35.3%, 115/326), the 20–39 years group (17.2%, 56/326), and those aged 80 years or older (10.1%, 33/326) (see Table 1).

**Table 1:** Epidemiologic features of the 326 selected cases

Sex	
Male	231
Female	95
Age	
< 20 years	4
21–40 years	57
41–60 years	117
61–80 years	115
> 80 years	33
BMI	
< 18.5 kg/m^2^	14
18.5–25 kg/m^2^	115
25–30 kg/m^2^	103
> 30 kg/m^2^	94
Time between corpse discovery and angiography *(six cases with unknown postmortem delay*)	
< 24 h	93
24-48 h	121
> 48 h	106
Known postmortem delay (*N* = 209)	
< 12 h	14
12-24 h	57
24-48 h	76
48-72 h	37
> 72 h	25
Radiologic Alteration Index (RAI)	
< 10	177
10–49	119
50–79	23
80–100	7
Cause of death	
Natural (including abdominal, cardiovascular, cerebral, infectious and metabolic origins and undetermined natural death)	201
Trauma (including car accident, fall, sharp trauma and gunshot wound)	89
Asphyxia (including hanging, smothering, choking, drowning and fire)	17
Intoxication	14
Death associated with surgical procedure	5

The delay between discovery of the body and performance of the MPMCTA was 24–48 h in 37% of cases (121/326), followed by more than 48 h (32.6%, 106/326) and less than 24 h (28.6%, 93/326). In six cases, the postmortem interval could not be determined (data not shown).

For more detailed statistical analysis, the actual postmortem interval (PMI) was calculated for cases in which the time of death was known (*N* = 209). Among these, the delay between death and MPMCTA was < 12 h in 7% (14/209), 12–24 h in 27% (57/209), 24–48 h in 36% (76/209), 48–72 h in 18% (37/209) and > 72 h in 12% (25/209) (see Table 1).

Regarding the causes of death, these were classified as follows:

- Natural causes (e.g. abdominal, cardiovascular, cerebral, infectious, metabolic origins, and undetermined natural deaths): 62% (201/326),
- Traumatic causes (e.g. stab wounds, gunshot wounds, traffic accidents, falls): 27% (89/326),
- Asphyxia (e.g. hanging, smothering, choking, drowning, fire-related deaths): 5% (17/326),
- Intoxication-related deaths: 4% (14/326),
- Deaths associated with surgical procedures: 2% (5/326) (see Table 1).

### Correlation between autopsy and angiography results

The study included 326 MPMCTAs performed prior to autopsy, among which 61% (199/326) showed positive intraluminal GI contrast agent extravasation classified as grade 3. These extravasations can easily be mistaken for hemorrhage by the untrained eye. In more than half of the grade 3 cases, contrast extravasation was observed at multiple locations: 59 cases with extravasation at two sites, 36 cases at three sites, and 10 cases at all four analyzed locations — the esophagus, stomach, duodenum, and small bowel. The most frequent site of extravasation was the stomach (*N* = 159) (see Table 2).

**Table 2:** Distribution and correlation of the positive cases between MPMCTA and autopsies

a. MPMCTA (*N* = 326)	200 (61%) positive for a grade 3 (either in the esophagus, stomach, duodenum and jejunum), or in several areas at once. Predominantly found in the stomach (*N* = 159)
	95 cases – 1 location
	59 cases – 2 locations
	36 cases – 3 locations
	10 cases – 4 locations
b. Autopsies (*N* = 326)	21 cases (6,7%) of GI hemorrhage (*7 causes of death*)
	17 cases show a correlation in location between MPMCTA/Autopsy grade 3 extravasations. In addition, all cases show grade 3 positivity in more than two phases (arterial and/or venous phases and dynamic phase) (cf. details in Table 3)
	2 cases showed only grade 1, one of which was not in the same location 2 cases were completely negative at the MPMCTA (false negative) (cf. details in Table 4)

a. Distribution of the number of locations in which extravasation is visible (grade 3) in cases of positive MPMCTA

b. Summary of the MPMCTA data in cases of autopsy-confirmed GI bleeding

GI hemorrhage was confirmed at autopsy in only 21 cases (6%) and was the cause of death in 7 cases (2%). Among these, six deaths were natural and secondary to upper GI hemorrhages (including two cases of esophageal varices, one duodenal ulcer, one esophageal rupture in the context of esophagitis, and two cases of diffuse gastric bleeding), and one was traumatic (esophageal rupture following nasogastric tube placement). Of these 21 cases, 17 were classified as true positives, defined as grade 3 extravasation with corresponding localization between MPMCTA and autopsy, although other sites of bleeding could also be present (see Tables 2 and 3

**Table 3:** Summary of positive cases on autopsy and grade 3 at MPMCTA (considered as « true positives»)

True positives				
		Cause of death	Site of bleeding at the autopsy	MPMCTA (grade 3)
Case 1	Woman, 45 yo	Upper gastrointestinal bleeding (exact origin unknown)	Intra gastric blood	Esophagus Stomach Duodenum Jejunum
Case 2	Woman 89 yo	Upper gastrointestinal bleeding following iatrogenic injury of the oesophagus	Intra esophagus blood	Esophagus Jejunum
Case 3	Man, 30 yo	Ruptured cerebral aneurysm	Intra gastric blood and traces of blood in the esophagus Slight erosion of the esophageal mucosa Few pinpoint hemorrhages of the gastric mucosa	Stomach Duodenum
Case 4	Woman, 74 yo	Post-anoxic encephylopathy after exposure to a fire	Intra gastric blood in presence of an ulcer Few pinpoint hemorrhages of the gastric mucosa	Stomach
Case 5	Woman, 43 yo	Cardiac tamponade due to ruptured aneurysm of the ascending aorta	Intra gastric “coffee ground” blood Pinpoint hemorrhages of the gastric mucosa	Stomach
Case 6	Man, 45 yo	Upper gastrointestinal hemorrhage due to ruptured esophageal varices	Intra esophagus and intra gastric blood Three slight erosions of the oesogastric junction mucosa Intra duodenal and jejunal “coffee ground blood"	Esophagus Stomach
Case 7	Woman, 77 yo	Cardiac tamponnade following intraoperative perforation	Intra jejunal blood (proximal part)	Duodenum
Case 8	Woman, 36 yo	Thoraco-abdominal gunshot	Penetrating wound of the stomach with intra gastric trace of blood Pinpoint hemorrhages of the gastric mucosa	Stomach Duodenum
Case 9	Man, 69 yo	Natural, undertermined	Intra gastric blood Pinpoint hemorrhages of the gastric mucosa	Stomach Duodenum Jejunum
Case 10	Woman 71 yo	Myocardial infarction	Intra gastric blood	Stomach Duodenum
Case 11	Man, 61 yo	Myocardial infarction	Intra gastric blood	Esophagus Stomach Duodenum Jejunum
Case 12	Woman, 50 yo	Cardiac decompensation	Intra gastric blood in presence of an ulcer	Stomach Duodenum Jejunum
Case 13	Woman, 57 yo	Upper gastrointestinal hemorrhage due to ruptured esophageal varices (exact origin not identified)	Distal esophagus	Esophagus Stomach
Case 14	Man, 46 yo	Fresh thrombosis of the circonflex artery and signs of myocardial ischemia	Intra gastric blood Hemorrhagic mucosa appearance	Stomach Duodenum
Case 15	Man, 47 yo	Upper gastrointestinal hemorrhage (exact origin not identified)	Intra gastric blood	Esophagus Stomach
Case 16	Man, 66 yo	Natural, undertermined	Intra gastric blood	Stomach
Case 17	Man, 62 yo	Upper gastrointestinal hemorrhage due to duodenal ulcer bleeding	Intra duodenal blood with ulcer	Esophagus Duodenum Jejunum

). Notably, all true positive cases except one showed contrast extravasation during the arterial or venous phases, not only during the dynamic phase. Based on these findings, we conducted a detailed analysis of the false positives to assess whether they more frequently exhibited extravasation exclusively during the dynamic phase. This hypothesis was not supported: one third of the cases demonstrated extravasation only during the dynamic phase, whereas two third of the cases also showed extravasation in the arterial and/or venous phases in addition to the dynamic phase. The remaining four cases included two with grade 1 mucosal enhancement and two with no angiographic signs (grade 0), all considered false negatives (see Table 5), including one fatal upper GI bleeding case (see Table 4)

**Table 4:** Summary of positive cases on autopsy and grade 0 (negative) or grade 1 at the MPMCTA (considered as « false negatives»)

False Negatives				
Case 18	Man, 66 yo	Fresh thrombosis of the left coronary artery	Intra esophagus and intra gastric blood	Stomach (grade 1) Duodenum (grade 1) Jejunum (grade 1)
Case 19	Man, 54yo	Myocardial infarction	Intra gastric blood Pinpoint hemorrhages of the gastric mucosa	Jejunum (grade 1)
Case 20	Man, 74 yo	Upper gastrointestinal hemorrhage due to laceration of the esophagus in the context of severe chronic esophagitis	Digested blood in the esophagus, stomach, duodenum and jejunum	Negative MPMCTA (grade 0)
Case 21	Man, 54 yo	Intoxication	Intra gastric blood Pinpoint hemorrhages of the gastric mucosa Several infracentimetric gastric ulcers	Negative MPMCTA (grade 0)

. These findings confirm a false positive angiographic extravasation rate of approximately 56% for grade 3 (182/326) (see Table 5).

**Table 5:** Summary of true and false negatives and true and false positives

	Positive MPMCTA (considered as grade 3)	Negative MPMCTA (considered as grade 0, 1 or 2)
Positive Autopsy	17 (true positive)	4 (false negative)
Negative Autopsy	182 (false positive)	123 (true negative)

Furthermore, 23 cases showed completely negative MPMCTA (grade 0) and no GI bleeding at autopsy. In most of these, death was due to massive non-GI hemorrhages (*N* = 21) of either natural origin (e.g., cardiac tamponade post-myocardial infarction or ruptured abdominal aneurysm) or traumatic origin.

No significant statistical correlation was found between artifactual contrast extravasation (grade 3) and age *(*< *or* > *60 years, Chi*^*2*^* p* = *0.35*), cadaveric Radiologic Alteration Index (RAI) *(*< *or* > *10, Chi*^*2*^* p* = *0.53*), or Body Mass Index (BMI) *(*< *or* > *25 kg/m*^*2*^*, Chi*^*2*^* p* = *0.15*) [22].

Interestingly, corpses with a postmortem interval (PMI) less than 24 h showed significantly more grade 3 extravasations compared to those with PMI greater than 48 h (*Chi*^*2*^* p* < *0.02*). Due to this unexpected result, cases with a precisely known time of death (*N* = 209) were re-analyzed, confirming a similar significant difference between PMI < 24 h (*N* = 71) and PMI > 48 h (*N* = 62) groups (*Chi*^*2*^* p* = *0.04*).

## Discussion

### Diagnostic elements for interpreting upper GI contrast extravasation on MPMCTA

**Fig. 1:**
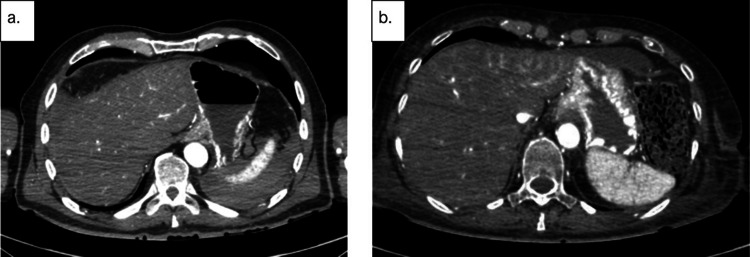
Axial views – Arterial phase. Image a. Enhancement of the gastric mucosa (grade 1). Image b. Enhancement and edema of the gastric mucosa (grade 2)

**Fig. 2:**
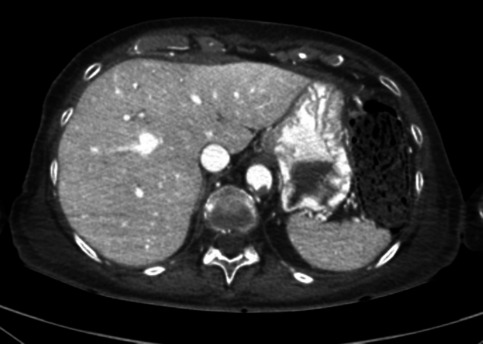
Axial view – dynamic phase. Diffuse extravasation of contrast medium in the gastric lumen (grade 3)

**Fig. 3:**
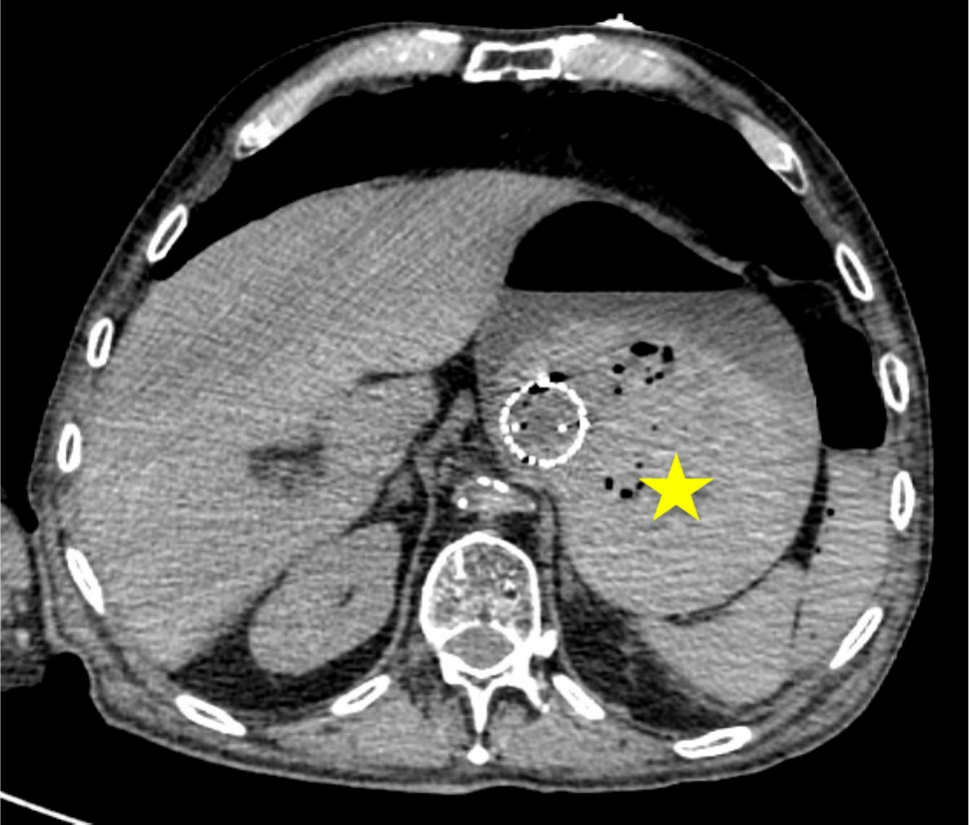
Axial view – native phase. Hyperdense material (yellow star) in the GI. lumen suggestive of blood in the native phase

Several patterns of angiographic GI contrast extravasation appear to follow a reproducible scheme and can assist in distinguishing true hemorrhage from artifacts. Based on our observations, we propose the following interpretative elements to support the radiologic assessment of upper GI findings on MPMCTA:

1. *Mucosal enhancement without intraluminal leakage is a nonspecific finding*. Homogeneous mucosal enhancement and edema (grades 1 and 2) should not be considered indicative of bleeding, but rather interpreted as aspecific postmortem changes (cf. Figure 1).
2. *The distribution pattern of contrast medium is essential*. When contrast is visible within the GI lumen (grade 3), the presence or absence of a clear focal point of leakage from the mucosa should be assessed. A diffuse and poorly localized extravasation suggests a likely artifact (cf. Figure 2).
3. *Temporal distribution across angiographic phases matters*. An extravasation visible only in the dynamic phase—and absent in the arterial and venous phases—should be considered non-specific and most likely postmortem in origin. The hypothesis suggesting that false positives were more likely to exhibit extravasation during the dynamic phase (than in arterial or venous phases previously) was not supported. However, in our study, the presence of extravasation exclusively during the dynamic phase demonstrated a negative predictive value of 94%. This reflects a general radiologic principle requiring a finding to be reproducible across phases to be considered significant [1].
4. *Careful inspection of the native scan can be informative*. In true upper GI bleeding, hyperdense material—interpretable as clotted blood—is often already visible on the unenhanced native scan, typically in dependent regions of the gastric lumen (cf. Figure 3).

**Fig. 4:**
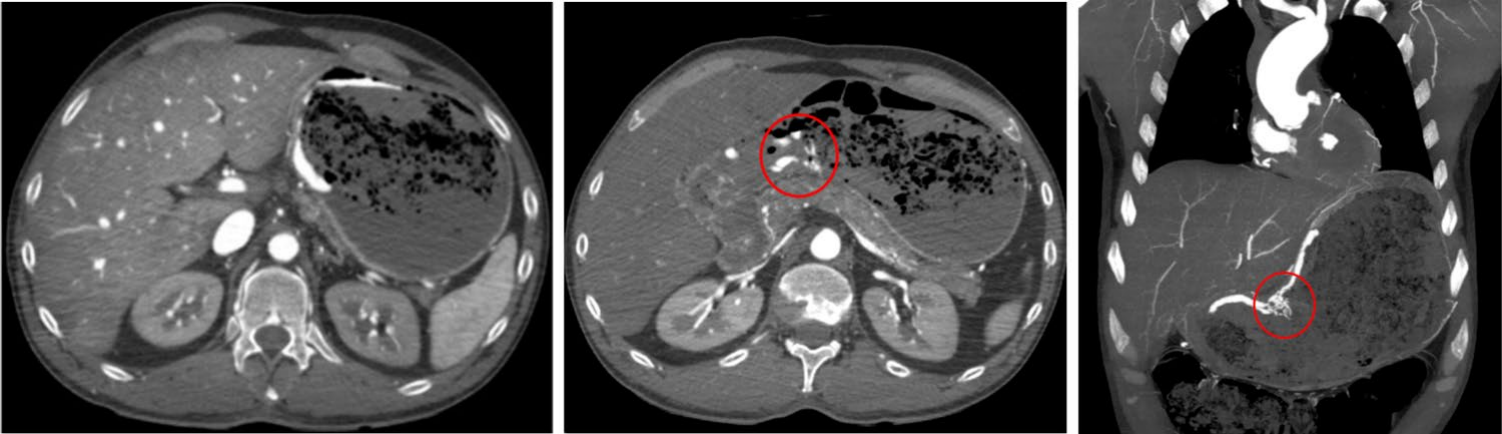
Axial views – (*from left to right*) dynamic phase – arterial phase – arterial phase. Focal extravasation of contrast medium suggesting gastric bleeding in the context of gastric ulceration (red circle)

**Fig. 5:**
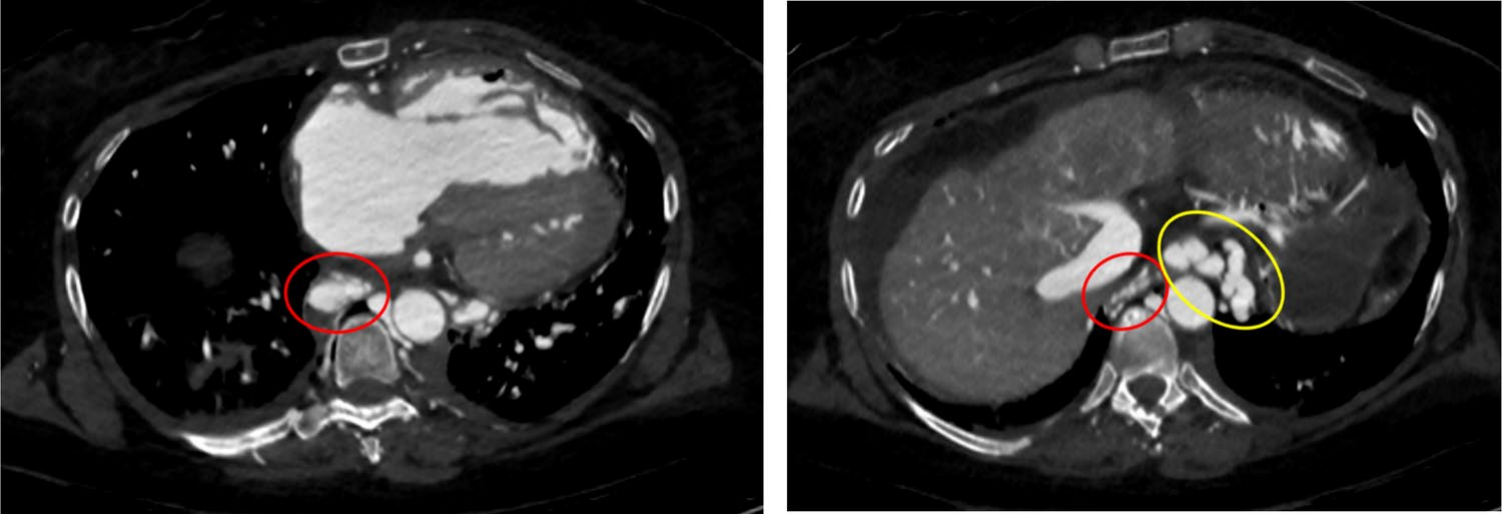
Axial views – Dynamic phase. Visible contrast medium in the esophageal lumen (red circle) and esophageal varices (yellow circle) highlighted in the dynamic phase of the MPMCTA suggesting esophageal bleeding in the context of ruptured esophageal varices

**Fig. 6:**
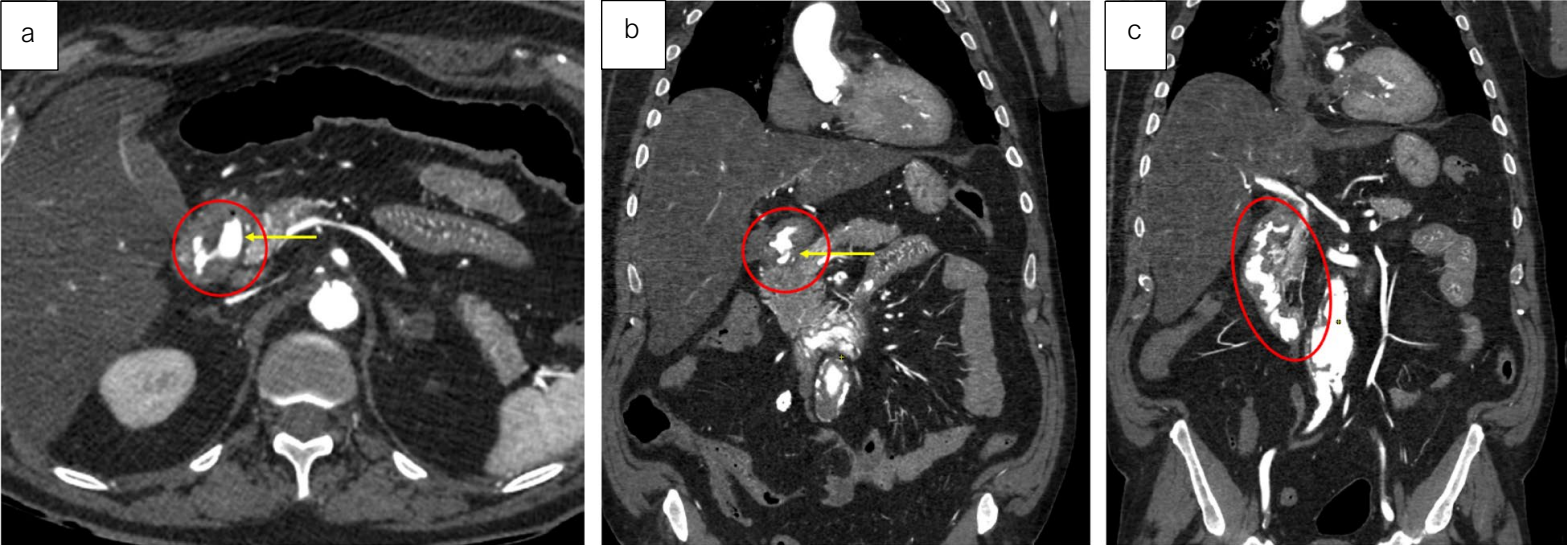
Arterial phase. Image **a** Axial view. Image **b** and **c**. Coronal views. Focal contrast medium extravasation in the context of duodenal ulceration (yellow arrow and red circle)

Therefore, true bleeding usually presents as focal and consistent extravasation. In confirmed hemorrhagic cases, a distinct, localized extravasation is generally seen during both arterial and/or venous phases. Examples from our study include gastric ulcer bleeding (cf. Figure 4), ruptured esophageal varices (cf. Figure 5) and duodenal ulcer bleeding (cf. Figure 6).

**Fig. 7:**
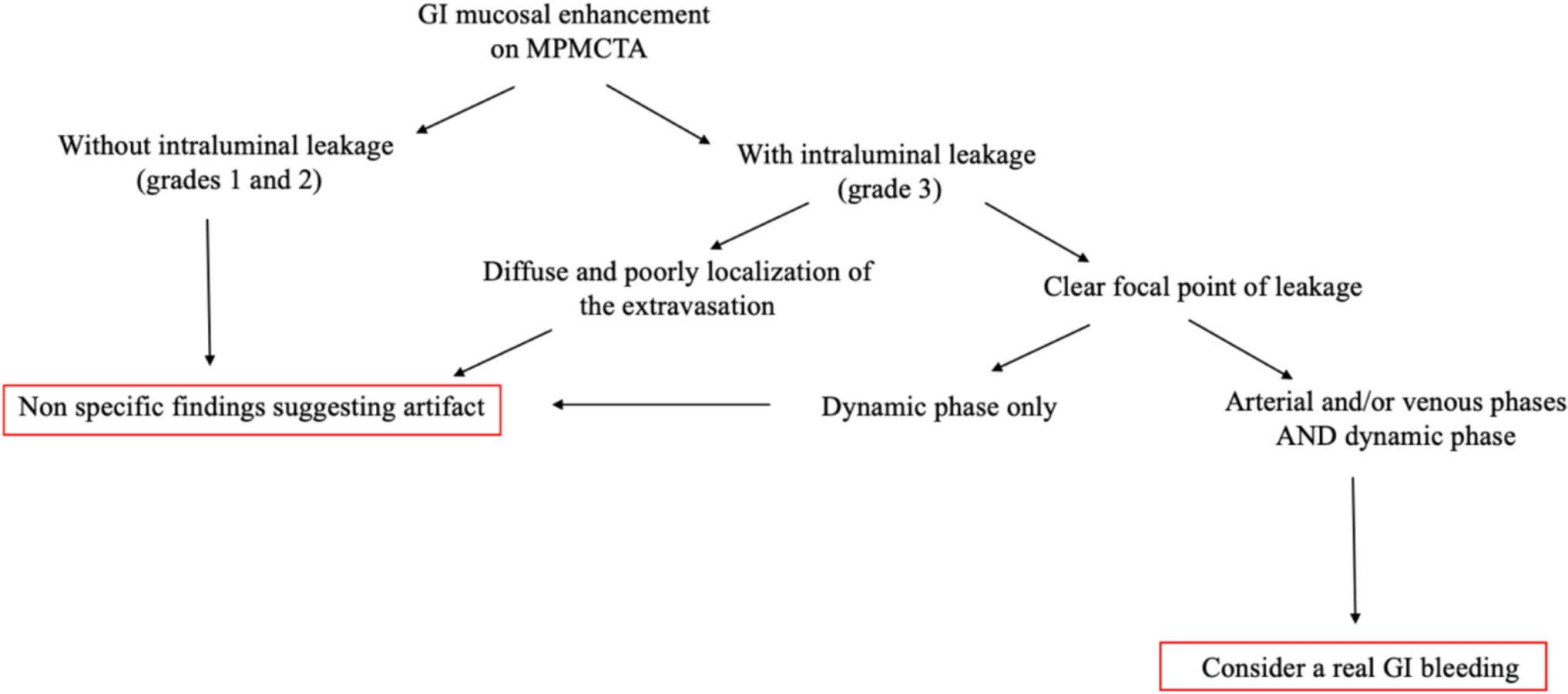
“Decision tree” to differentiate whether it is most likely an artifactual origin or a real GI bleeding

These elements are summarized in Table 6 and in a “decision tree” (cf. Figure 7) and may serve as a support tool for radiologic interpretation in forensic settings.

**Table 6:** Diagnostic tips for the interpretation of contrast product extravasation in MPMCTA

True positive	Not significant	Angiographic artifact (false positive)
Hyperdense material in the GI lumen in the native phase Focal extravasation of contrast product Extravasation visible during arterial or venous phases (not only dynamic phase)	Amount of contrast product	Diffuse enhancement of the mucosa (grade 1 or 2) Extravasation of contrast product without any focal leak of contrast agent Extravasation of contrast product during the dynamic phase only

### Interpretation of other findings

In recent years, forensic radiological investigations have significantly enhanced the diagnostic capabilities in postmortem examinations, with MPMCTA now considered the gold standard for detecting sources of hemorrhage. However, diagnosing GI tract hemorrhage postmortem remains challenging due to the presence of technical artifacts. Currently, there are no established guidelines to interpret angiographic extravasations in the GI tract, which complicates diagnosis.

The primary objective of our study was to evaluate the usefulness of MPMCTA in detecting and localizing the source of GI bleeding, whether fatal or not. Despite the high diagnostic potential of this imaging modality, limitations exist, notably a high artifact rate, which was nearly 56% false positives (grade 3 extravasation) in our cohort. Most extravasations occurred in the stomach, a finding consistent with the organ’s high concentration of hydrolytic enzymes and anatomical proximity to the pancreas. These findings should be considered routine artifacts by forensic radiologists and distinguishing them from true bleeding requires specific diagnostic criteria, as discussed above. Importantly, radiologic interpretation must always be integrated with clinical and forensic information provided by the pathologist.

Approximately two-thirds of cases were deaths from natural causes. This is expected since our institute performs MPMCTA routinely in suspected cardiovascular deaths. Conversely, MPMCTA is usually avoided in cases of severe trauma without signs of life at emergency arrival, to enable other examinations such as fat embolism detection as signs of vitality. In our study, upper GI bleeding accounted for only 3% of natural deaths examined by angiography, a rate almost ten times lower than that reported by colleagues from India. This difference likely reflects the high proportion of forensic autopsies performed at our center (about 5% of all deaths), leading to a larger share of natural deaths in the sample.

An unexpected finding was that corpses with a postmortem interval (PMI) less than 24 h exhibited significantly more grade 3 extravasations. To explore this, we reanalyzed cases with precisely known times of death (*N* = 209, mostly in-hospital deaths with documented resuscitation) and confirmed the statistical significance of this result. This observation aligns with the experience reported in the *Atlas of Postmortem Angiography*, who noted that “*Concerning artefacts caused by postmortem changes, our experience shows that extravasations into the gastrointestinal tract are less important than might be expected*” [17]. Our working hypothesis is that relatively well-preserved mucosal tissue is necessary for the contrast enhancement to occur. Early postmortem autolysis — the enzymatic destruction of cells and tissues— may increase mucosal permeability, allowing contrast extravasation through microlesions during the initial stages of decomposition [23]. It is important to remember that in case of artifactual extravasation of contrast medium, it is the autolysis process that will have an influence. Indeed, the putrefaction process, linked to the migration of bacterial flora through the blood vessels, will have no impact on the extravasation of contrast medium. Therefore, it seems logical that the RAI has no statistical correlation with the false positive rate but only on gaz formation [24].

Regarding negative cases (grade 0), most had massive hemorrhages as the cause of death identified at autopsy, but these were non-GI in origin. Only one case was a false negative for upper GI bleeding. This suggests that massive bleeding may cause contrast medium to leak elsewhere, reducing the occurrence of artifactual GI extravasations. In the false negative case, involving esophageal rupture in the setting of severe chronic esophagitis, we hypothesize that a blood clot near the lesion may have prevented contrast extravasation and subsequent detection.

### Limitations of the study

One main limitation of this study lies in the relatively low number of deaths caused by upper GI hemorrhage among the overall cases examined. This small sample size may limit the statistical power of our findings when evaluating the diagnostic performance of MPMCTA specifically for fatal upper GI bleeding. Further studies involving larger cohorts or multicenter collaborations would be useful to confirm and refine the proposed interpretative elements.

Moreover, another limitation of our study is that we used a single, oil-based contrast agent mixture. As a result, our findings may not be generalizable to cases involving other types of contrast agents, particularly water-soluble mixtures.

## Conclusion

In contrast to clinical radiology, where contrast extravasation is typically interpreted as a sign of active bleeding, postmortem angiographic imaging requires cautious interpretation due to known artifacts associated with postmortem changes and technical aspects of perfusion protocols [18].

In particular, contrast extravasation in the upper GI tract should not be automatically equated with true hemorrhage. When GI bleeding is confirmed at autopsy, certain radiologic features on MPMCTA—such as hyperdense intraluminal material on the native scan and a focal site of contrast leakage during the arterial or venous phase—may be indicative of true bleeding.

The diagnostic elements identified in our study could assist radiologists and forensic pathologists in interpreting MPMCTA in this context, enhancing the diagnostic utility of the method while acknowledging its limitations.

## Data Availability

The data are available from the corresponding author on reasonable request.
